# Continuous multidimensional physiological monitoring redefines early warning and preemptive treatment for neutropenic sepsis following cisplatin chemotherapy in non-small cell lung cancer

**DOI:** 10.3389/fphar.2026.1861637

**Published:** 2026-08-21

**Authors:** Zhe Wang, Kunpeng Yang, Lei Wang, Hui Zhao, Peiyun Lv, Chenglun Cai, Wenjuan Sun, Bao Wang

**Affiliations:** 1 Department of Thoracic Oncology Surgery II, Jilin Cancer Hospital, Changchun, China; 2 School of Clinical Medicine, Changchun University of Chinese Medicine, Changchun, China

**Keywords:** cisplatin, continuous physiological monitoring, explainable artificial intelligence, neutropenic sepsis, non-small cell lung cancer

## Abstract

**Background:**

Despite the advent of targeted therapies, cisplatin remains a cornerstone in the management of non-small cell lung cancer Its systemic toxicity, however, often triggers neutropenic sepsis, a rapidly fatal oncological emergency. Traditional intermittent monitoring is inadequate here. Chemotherapy-induced “inflammatory silence” frequently masks the classical febrile response, leaving patients unprotected during outpatient windows.

**Methods:**

In this Perspective, we mined global real-world pharmacovigilance data from the FAERS and Japanese Adverse Drug Event Report databases. We applied four disproportionality-analysis algorithms and Weibull time-to-onset modeling to reconstruct the systemic toxicity spectrum and temporal risk trajectory of cisplatin. We then synthesize the conceptual and technical basis for continuous multidimensional physiological monitoring (cVSM) coupled with explainable artificial intelligence (XAI).

**Results:**

We reveal a distinct “early failure” mode (Weibull shape parameter β = 0.81) for severe cisplatin-related adverse events, clustered within the first 30 days after infusion. We also show that threshold-based alerts tend to induce “alarm fatigue”, because the physiological baselines of cancer patients shift dynamically, as in sarcopenia-driven occult overexposure in older patients. To address this, we propose an XAI-driven predictive framework integrated with non-invasive point-of-care testing The framework decodes subtle autonomic changes, such as reduced heart rate variability, hours before overt septic shock.

**Conclusion:**

cVSM coupled with XAI can shift the management of chemotherapy-induced toxicity from reactive rescue to pre-emptive therapeutics. Realizing this digital safety net will require revised oncology monitoring guidelines, restructured reimbursement models, and the deployment of decentralized clinical trials.

## 1 Introduction

The disease burden of non-small cell lung cancer (NSCLC) remains high (Zhang et al., 2025). According to the 2021 Global Burden of Disease (GBD) study, tracheal, bronchus, and lung (TBL) cancers caused 2.28 million incident cases and 2.04 million deaths worldwide in 2021, with 45.90 million disability-adjusted life years (DALYs) (Liu et al., 2025). Over recent decades, age-standardized mortality rates and age-standardized DALY rates have declined modestly. These gains were driven mainly by global tobacco control and by the wider use of low-dose computed tomography (LDCT) screening. Yet they are increasingly offset by global population aging (Zhang et al., 2025). GBD 2021 data show that the age-standardized incidence rate among adults aged 70 years or older reached 225.78 per 100,000, far above the 15.74 per 100,000 seen in younger people. This gap points to a continued expansion in the absolute burden of lung cancer (Liu et al., 2025).

Systemic therapy for NSCLC has advanced through targeted agents, such as EGFR inhibitors, and immune checkpoint inhibitors, including PD-1 agents. Even so, platinum-based doublet chemotherapy, particularly cisplatin, remains a cornerstone of treatment (Shimokawa et al., 2021). Cisplatin forms intrastrand and interstrand cross-links with tumor-cell DNA. This impairs DNA templating and, through reactive oxygen species–mediated DNA damage, potently induces apoptosis in lung cancer cells (Mohiuddin and Kasahara, 2021).

As a heavy-metal complex, cisplatin has a complex pharmacokinetic profile, wide tissue distribution, and marked cytotoxicity. This non-specific cytotoxicity targets malignant cells with high proliferative activity. It also affects normal tissues with rapid turnover, such as bone marrow hematopoietic stem cells and gastrointestinal mucosal epithelium (Mohiuddin and Kasahara, 2021; Liu W. et al., 2024). As a result, severe multi-system toxicity, notably dose-limiting myelosuppression, remains a key bottleneck that constrains both quality of life and long-term prognosis in NSCLC (Lyu et al., 2025).

Among chemotherapy-related adverse events, neutropenic sepsis from severe myelosuppression is one of the most unpredictable and lethal oncological emergencies (Dracham et al., 2025; Taplitz et al., 2018). International guidelines, including those from the IDSA, ASCO, and ESMO, define strict diagnostic criteria (Klastersky et al., 2016; Freifeld et al., 2011). The biochemical threshold is an absolute neutrophil count (ANC) below 500 cells/μL. It also applies when the current ANC is below 1,000 cells/μL but is projected to fall below 500 cells/μL within 48 h. For thermoregulation, fever is classically defined as a single oral or core temperature of ≥38.3 °C (101°F). It is also diagnosed when a temperature of ≥38.0 °C (100.4°F) persists for more than 1 h under continuous monitoring.

In patients with NSCLC whose immune defenses are severely compromised, even low-virulence opportunistic pathogens can drive an uncontrolled systemic inflammatory response syndrome (SIRS) within hours. This often progresses rapidly to irreversible septic shock and multiple organ dysfunction syndrome (MODS) (Cheng et al., 2024; Xu et al., 2025). Epidemiological and critical-care data show a very high short-term mortality in severe neutropenic sepsis. Survival is inversely related to the “door-to-needle time”. Each hour of delay in broad-spectrum antibiotics raises mortality risk sharply (Philippon et al., 2025).

Reliance on these classical criteria alone, however, can overlook the pathophysiology seen in the clinic and lead to diagnostic pitfalls. Potent agents such as cisplatin cause severe leukocyte depletion, which often impairs the production of endogenous pyrogens, including interleukin-1 (IL-1), interleukin-6 (IL-6), and tumor necrosis factor-α (TNF-α) (Clarke et al., 2013). Through this “inflammatory silence”, the early clinical signs of sepsis are blunted or masked, especially the classical high-fever threshold of ≥38.3 °C (Forde and Scullin, 2017).

Outpatient monitoring blind spots, which often last days or weeks, combine with the silent onset of sepsis. Together they form the core limitation of intermittent thermometry. This flaw motivates continuous multidimensional physiological monitoring (cVSM) systems. The value of cVSM lies not only in moving beyond a single temperature threshold. More importantly, it can capture subtle pathophysiological changes early in disease evolution. Specifically, it can detect micro-fluctuations in heart rate variability (HRV) and compensatory rises in resting heart rate caused by autonomic dysfunction.

**FIGURE 1:**
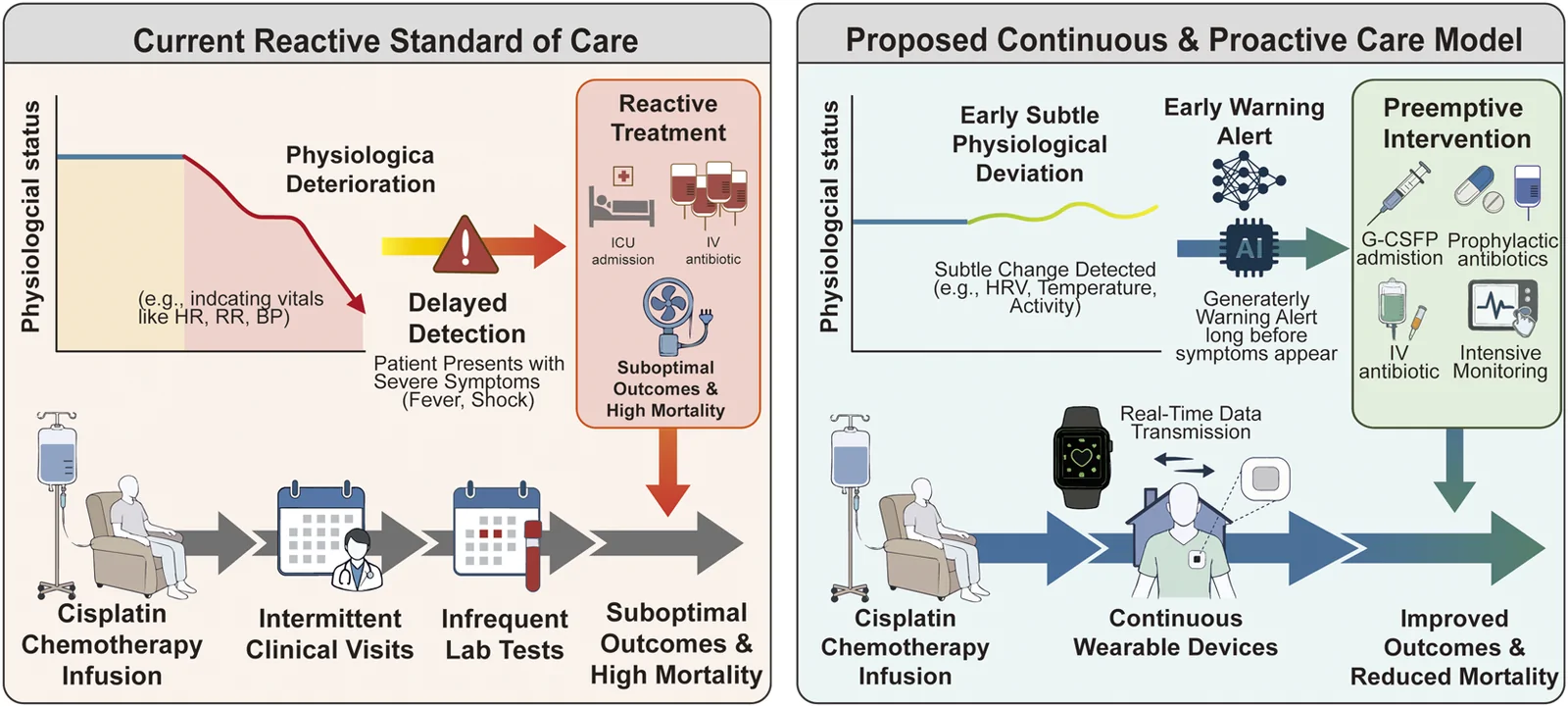
The Reactive-to-Proactive Shift in Neutropenic Sepsis Management in NSCLC Patients. Conceptual overview contrasting the current reactive standard of care (left panel) with the proposed continuous, proactive care model (right panel) for non-small cell lung cancer (NSCLC) patients undergoing cisplatin chemotherapy. The main goal is to shift from symptom-driven treatment to pre-symptomatic detection of physiological deterioration through continuous monitoring. (Left Panel) Current Reactive Standard of Care. This model relies on intermittent monitoring (calendar icons) following cisplatin chemotherapy infusion. Patients return for scheduled, Intermittent Clinical Visits and Infrequent Lab Tests, leaving long monitoring gaps. (Top Left Graph) The graph of Physiological Status shows a sharp red decline indicating rapid deterioration that is not detected during the pre-symptomatic phase. (Center Left Flow) Detection is delayed until the patient self-reports with severe symptoms like fever or shock, marking Delayed Detection. This leads to reactive, high-cost, and high-intensity clinical responses (Reactive Treatment), including ICU admission and multiple intravenous antibiotics, often resulting in Suboptimal Outcomes and High Mortality. (Right Panel) Proposed Continuous and Proactive Care Model. This model integrates multi-dimensional data from Continuous Wearable Devices (e.g., smartwatch, chest patch sensor) immediately following cisplatin chemotherapy, allowing for Real-Time Data Transmission while the patient is at home. (Top Right Graph) The graph shows that Early Subtle Physiological Deviation (e.g., changes in heart rate variability, temperature, or activity patterns) is detected long before standard vital signs fail. (Center Right Flow) An Artificial Intelligence (AI) algorithm processes this fused data stream in real-time, generating an Early Warning Alert during the pre-symptomatic phase. This triggers immediate Preemptive Intervention, enabling personalized treatment decisions (e.g., targeted diagnostics, G-CSF administration, prophylactic antibiotics, and intensive monitoring), ultimately leading to Improved Outcomes & Reduced Mortality.

Building on this rationale, and grounded in large-scale real-world data (RWD) combined with modern artificial intelligence, this Perspective proposes a shift in view: cVSM should no longer be defined narrowly as a digital extension of traditional vital-sign recorders. In the era of digital health and precision medicine, cVSM serves as the core digital infrastructure for closing the outpatient monitoring blind spot. It drives a shift in the management of lethal post-chemotherapy complications, from reactive rescue to pre-emptive therapeutics. By reconstructing the spatiotemporal distribution of cisplatin toxicity and integrating explainable artificial intelligence (XAI), cVSM builds a digital safety net for patients with NSCLC and redefines the standards of pharmaceutical and clinical care for oncological emergencies (Figure 1).

## 2 RWD mining: reconstructing the systemic characteristics and spatiotemporal distribution of cisplatin toxicity

Traditional randomized controlled trials (RCTs) are limited by small sample sizes, short follow-up, the exclusion of complex comorbidities, and strict enrollment criteria. It is therefore important to mine the features of cisplatin-induced adverse events (AEs) in complex real-world settings (Ye et al., 2025). In this study, cisplatin-related cases were extracted from the US FDA Adverse Event Reporting System (FAERS; Q1 2004 to Q3 2024) and the Japanese Adverse Drug Event Report (JADER) database (Q1 2005 to Q2 2024).

To reduce heterogeneity and reporting bias, we applied a strict data-cleaning and standardization protocol. Duplicated and logically anomalous records were removed per official guidelines, and cases lacking essential demographic data were excluded. All AEs were then mapped to the Preferred Term (PT) and System Organ Class (SOC) levels using the MedDRA 26.0 dictionary (Hai et al., 2024).

For signal identification and spatiotemporal modeling, we applied four disproportionality-analysis (DPA) algorithms based on a 2 × 2 contingency table (Supplementary Table S1). These algorithms, the Reporting Odds Ratio (ROR), Proportional Reporting Ratio (PRR), Multi-item Gamma Poisson Shrinker (MGPS), and Bayesian Confidence Propagation Neural Network (BCPNN), were used for signal extraction and dual-database cross-validation (models and thresholds in Supplementary Table S2) (Park et al., 2022). In parallel, reports with complete drug-administration and event-onset dates were extracted to calculate the time-to-onset (TTO). A Weibull distribution model was then fitted to the dynamic risk trajectories. This data architecture provides an evidence-based foundation for analyzing the true toxicity spectrum of cisplatin in complex clinical settings (Cui et al., 2023).

### 2.1 Comparative analysis of regional and global demographic profiles

Before signal mining, defining the baseline demographic profile of the cohorts is a prerequisite for external validity. Among the 995 core cisplatin-related AE reports from FAERS, male patients predominated (54.2%), while females accounted for 28.7%. The age distribution skewed younger, with patients aged <65 years at 46.0% versus 30.1% for those ≥65 years. Geographically, reports were mostly from developed Western nations (United States 33.9%, Germany 12.5%, United Kingdom 7.1%).

By contrast, the 651 core reports from JADER, which reflect domestic Japanese data, showed a stronger gender skew: male patients reached 72.8%, while females were only 24.1%. In both databases, most AEs were reported by healthcare professionals (70.3% in FAERS and 98.4% in JADER). This high share of professional reporting supports the accuracy and clinical reliability of the downstream signal extraction. These structural differences in gender and geography also reflect possible pharmacogenomic differences between East Asian and Caucasian populations, both in the epidemiology of NSCLC and in tolerance to cisplatin-based chemotherapy.

### 2.2 Cross-validation of SOC safety signals and shock warning

In pharmacovigilance and disease early warning, signal detection from a single database carries uncertainty because of reporting bias. Bidirectional cross-validation between FAERS and JADER raises statistical power and integrates multi-ethnic safety data, which improves the reliability of the results.

At the SOC level (Supplementary Tables S3, S4), the strongest FAERS toxicity signals were in Blood and Lymphatic System Disorders (ROR 2.51, positive across all four algorithms), Vascular Disorders (ROR 2.16), and Gastrointestinal Disorders, the last being the most frequent. In JADER, Vascular Disorders was the strongest signal (ROR 3.78), followed by Blood and Lymphatic System Disorders (ROR 2.54). This consistency across regional and ethnic databases validates the risk signals epidemiologically. It also points to the underlying pathophysiology of cisplatin injury: beyond the bone-marrow microenvironment (hematopoietic stem-cell exhaustion), it involves microvascular endothelial damage and microcirculatory impairment.

A more granular PT-level analysis highlights the lethal consequences of these systemic toxicities. In FAERS, “Neutropenic sepsis” showed very high signal intensity (ROR 9.23). Notably, “Septic shock”, a critical event often overlooked in practice, was cross-validated in both databases (FAERS ROR 4.76; JADER ROR 5.24) (Table 1). These consistent signals indicate that cisplatin-induced severe myelosuppression tends to evolve into systemic inflammatory response syndrome (SIRS) and hemodynamic failure. The rapid progression from occult infection to irreversible shock is thus an established clinical trajectory, not a coincidence.

**TABLE 1:** Real-world signal intensities of cisplatin-induced neutropenic sepsis and septic shock.

Database source	Preferred term (PT)	Number of cases (n)	Reporting Odds Ratio (ROR) (95% CI)	Proportional Reporting Ratio (PRR)
FAERS	Neutropenic sepsis	26	9.23 (6.16 - 13.83)	9.15
FAERS	Septic shock	22	4.76 (3.09 - 7.31)	4.73
JADER	Septic shock	11	5.24 (2.78 - 9.90)	5.21

### 2.3 The “early failure” pattern: the critical temporal risk quadrant revealed by weibull distribution

Defining the TTO of adverse events and building dynamic evolutionary models are pivotal for time-based digital health monitoring. After survival and risk modeling of 482 valid TTO reports from FAERS, the data showed consistent temporal patterns. The toxicity onset of cisplatin closely fits the Weibull distribution used in reliability engineering. The fit gave a scale parameter (α) of 41.94 (95% CI: 37.05–46.83) and a shape parameter (β) of 0.81 (95% CI: 0.76–0.86). In survival analysis, β determines the risk trajectory: β = 1 indicates random failure, and β > 1 indicates wear-out failure. The value β = 0.81 indicates a classic “early failure” pattern, meaning the hazard function decreases over time.

**FIGURE 2:**
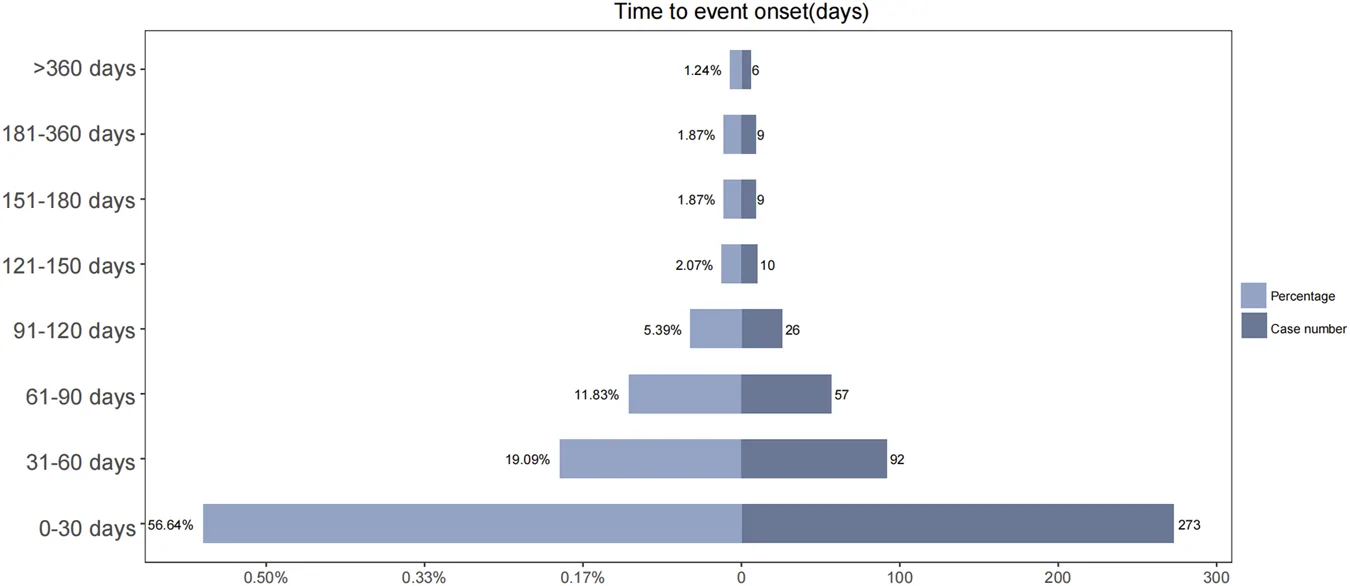
Time to onset (TTO) of Cisplatin-induced adverse events.

Real-world data support this deduction. Among severe systemic adverse events induced by cisplatin (Figure 2; Table 2), 56.64% occurred within the first 30 days after treatment initiation, with a median TTO of 23 days (interquartile range [IQR]: 9–59 days). This 30-day high-risk window aligns with the kinetics of myelosuppression. The neutrophil nadir typically occurs between days 7 and 14 after chemotherapy, when impaired innate immune defenses sharply raise infection risk (Melhem et al., 2018). Within this maximum-risk quadrant, the standard follow-up schedule, usually every 21 or 28 days, fails to capture transient clinical fluctuations. This creates a substantial monitoring lag and a critical risk blind spot.

**TABLE 2:** Time to onset of Cisplatin-associated adverse events and Weibull distribution analysis.

Drug	TTO (days)		Weibull distribution		
Cisplatin	Case reports	Median(d) (IQR)	Scale parameter: α (95%CI)	Shape parameter: β (95%CI)	Type
	482	23 (9-59)	41.94 (37.05-46.83)	0.81 (0.76-0.86)	Early failure

Abbreviation: TTO, time to onset; CI, confidence interval; IQR, interquartile range.

Targeting the 30-day post-chemotherapy window with cVSM is therefore a clinical necessity. Wearable sensors enable continuous 24-h thermometry to detect blunted low-grade fevers. More importantly, high-frequency sampling can capture subtle declines in HRV, compensatory rises in resting heart rate, and small changes in respiratory rate. Critical-care research has repeatedly shown that autonomic dysfunction, seen as a marked HRV decline, often precedes overt high fever or hypotensive shock by hours or days. This dysregulation is driven by early septic inflammatory cytokines that inhibit the vagal nerve (Liu X. et al., 2024). Using continuous multidimensional monitoring to capture these subclinical signals gives a strong rationale for high-density monitoring in the first month after chemotherapy. It also forms the core digital strategy for reversing high mortality and achieving the shift from reactive rescue to pre-emptive therapeutics.

Taken together, the macro-epidemiological signals mined from FAERS and JADER do more than quantify the systemic toxicity burden of cisplatin; they define, in objective temporal and organ-system terms, precisely where and when a monitoring solution must operate. The convergence of a cross-validated septic-shock signal, a male- and elderly-skewed susceptibility profile, and a Weibull-defined 30-day Critical Temporal Risk Quadrant collectively delineates a concrete surveillance target that episodic outpatient follow-up structurally cannot meet. This transition from population-level risk mapping to individual-level continuous surveillance is the logical bridge that motivates the remainder of this Perspective: in the following section, we translate these database-derived trends into the concrete deployment of wearable sensor technology and explainable artificial intelligence at the point of care.

## 3 Clinical challenges and opportunities in digital early warning

Continuous physiological monitoring devices, such as flexible patches, smartwatches, and in-ear monitors, have advanced in hardware and data acquisition. Even so, simple threshold-based alert systems struggle in the complex, real-world setting of medical oncology (Rama et al., 2025). To turn these capabilities into clinical value, we must integrate deeper pathophysiological insight, better risk stratification, and explainable artificial intelligence (XAI) algorithms.

### 3.1 Highly complex physiological baselines and clinical “alarm fatigue”

During cisplatin-based chemotherapy, the physiological baseline of patients with NSCLC is not a static steady state. Instead, it undergoes marked internal perturbations. Beyond myelosuppression, the gastrointestinal and renal toxicity of cisplatin adds further physiological challenges. RWD from FAERS show that nausea (n = 63, ROR = 1.56) and vomiting (n = 62, ROR = 2.08) are the most frequent gastrointestinal adverse events. Severe vomiting often causes marked hypovolemia and isotonic dehydration. In addition, up to 30% of patients receiving cisplatin develop acute kidney injury (AKI) of varying degree, which leads to severe electrolyte imbalance, such as hyponatremia and hypokalemia.

Through compensatory mechanisms, dehydration, hypovolemia, or electrolyte imbalance usually raises resting heart rate (compensatory tachycardia) and causes blood-pressure fluctuations (Wei et al., 2025). For conventional rule-based algorithms or early-warning scores such as qSOFA and MEWS, these vital-sign changes from severe chemotherapy side effects are almost indistinguishable, on numerical parameters alone, from the hemodynamic shifts of early neutropenic sepsis, namely vasodilation and tachycardia (Gull et al., 2020; Lind et al., 2021).

If clinical decision support systems (CDSS) or monitoring algorithms cannot extract oncology-specific pathophysiological features, they tend to trigger many false-positive alarms in the first week after chemotherapy. This yields a very low positive predictive value (PPV). Such frequent invalid alerts raise the cognitive load on oncology staff and induce “alarm fatigue”, a key concern in human-factors engineering. As teams grow desensitized and habitually dismiss high-frequency alarms, the subtle prodromal signals of early hemodynamic instability in sepsis are lost to “signal masking”. The result is severe decision latency, which strips the monitoring system of its core clinical value.

### 3.2 Subgroup bias and risk stratification based on real-world evidence

To reduce alarm fatigue and improve the specificity of early warnings, we must translate individual baseline risks into prior probabilities and embed them in the monitoring algorithms. Subgroup analyses of FAERS and JADER show a strong link between demographic features and susceptibility to cisplatin toxicity, which supports the development of precise risk stratification models.

Our subgroup analyses show that male and elderly (≥65 years) patients with NSCLC are more susceptible to myelosuppression and renal injury. In this group, infectious pneumonia, severe anemia, thrombocytopenia, AKI, and septic shock cluster strongly. This pattern is not merely demographic; it calls for analysis at the micro-pharmacokinetic (PK) level. In routine practice, cisplatin dosing is calculated from body surface area (BSA), yet its systemic safety and clearance depend on the true glomerular filtration rate (GFR) (Arora et al., 2026). GFR is widely estimated from serum creatinine using the Cockcroft-Gault or MDRD formulas.

However, creatinine is a direct byproduct of skeletal muscle metabolism. In the specific subgroup of elderly male patients with NSCLC, the combined effects of long-term smoking, cancer cachexia, and the natural aging process frequently result in a high prevalence of age-related sarcopenia. Due to a substantial reduction in total muscle mass, baseline creatinine production in these high-risk patients may be correspondingly decreased. This creates a deceptive phenomenon where serum creatinine levels appear to remain within “normal” or “low” ranges. Consequently, formulaic calculations based on these low or spuriously normal creatinine levels harbor a significant risk of overestimating the patient’s true renal excretory function (Mathe et al., 2011). When standard doses of cisplatin are administered based on this potentially overestimated GFR, the actual *in vivo* clearance may be insufficient to effectively metabolize the drug, thereby increasing the likelihood of excessive area under the curve (AUC) and prolonged plasma exposure (Klockl et al., 2020). This insidious drug overexposure—driven in part by sarcopenia—coupled with the irreversible immunosenescence characteristic of the elderly population, is considered a critical mechanism triggering profound hematopoietic stem cell toxicity (i.e., severe myelosuppression) and acute tubular necrosis (Prado et al., 2009).

Conversely, female and younger (<65 years) patients face a higher frequency of gastrointestinal hypersensitivity (e.g., severe nausea, esophagitis, mucosal inflammation) and more insidious thromboembolic events (e.g., pulmonary embolism, aortic thrombosis). Younger patients may be more prone to nausea due to receiving higher dose-density chemotherapy regimens and exhibiting more robust inflammatory cytokine release responses (Navari and Aapro, 2016). Females, owing to differences in volume of distribution and the potential interference of estrogen with drug-metabolizing enzymes, are more predisposed to manifesting vascular toxicity and gastrointestinal intolerance (Aljohmani and Yildiz, 2026). This toxicity bias—determined by sex-based pharmacokinetic differences, cancer-induced wasting states, and age-related physiological baselines—demands that digital monitoring systems abandon “one-size-fits-all” warning thresholds in favor of highly specific, adaptive parameter modulation capabilities (Wagner et al., 2019).

### 3.3 Precision early warning driven by explainable artificial intelligence (XAI)

Given the complex baselines and diverse subgroups of chemotherapy patients, integrating cVSM with RWD-based risk stratification, together with explainable AI (XAI), is a key opportunity to overcome technical bottlenecks and rebuild clinical trust (Abbas et al., 2025).

In traditional decision support, deep-learning models often meet clinical resistance because of their “black-box” nature. In critical-care early warning, physicians need more than a risk probability; they must understand why the algorithm makes its prediction. XAI changes this. By building Dynamic Bayesian Networks (DBNs) or using tree-based ensembles explained with SHAP (SHapley Additive exPlanations) values, predictive systems can combine causal-inference logic with multimodal data (Agard et al., 2025).

Architecturally, the XAI model first extracts baseline features from electronic health records (EHRs). These include age, sex, baseline hepatic and renal function, skeletal muscle mass (if available), exact cisplatin dose, and concurrent medications, such as beta-blockers or other chronotropic agents. The system then ingests real-time, continuous time-series data from cVSM, including heart rate, body temperature, respiratory rate, and oxygen saturation. While computing the risk probability, the model also generates transparent attribution pathways (Li et al., 2024).

Crucially, the fidelity of these baseline features hinges on an accurate estimate of true renal function, and here the choice of biomarker is decisive. Unlike creatinine (whose generation is a direct function of skeletal muscle mass, sex, and dietary status), Cystatin C is a low-molecular-weight protein produced at a near-constant rate by all nucleated cells, rendering it largely independent of muscle mass and body composition. This distinction is not academic in the sarcopenic, elderly, male NSCLC population identified above, in whom creatinine-based equations systematically overestimate the glomerular filtration rate (GFR) and thereby licence occult cisplatin overexposure. Consistent with the 2024 KDIGO clinical practice guideline for CKD and the position statement of the American Society of Onco-Nephrology, single creatinine-based estimating equations should be abandoned in patients with cancer, particularly those with suspected sarcopenia, in favour of the combined CKD-EPI 2021 creatinine–cystatin C equation (Kidney Disease: Improving Global Outcomes KDIGO CKD Work Group, 2024; Kitchlu et al., 2024). This distinction carries a direct dosing consequence: because carboplatin is dosed to a target area-under-the-curve via the Calvert formula, in which the estimated GFR enters the dose calculation directly, reliance on the Cockcroft-Gault formula alone can dictate a carboplatin dose ≥25 mg higher than that derived from the CKD-EPI equation, and patients subject to this discrepancy carry a significantly elevated risk of grade ≥2 adverse events and chemotherapy discontinuation (Lyu et al., 2025). Concordantly, across validated equations the Cockcroft-Gault formula produces the largest carboplatin-dosing error, exceeding 20% in more than one-quarter of patients (Kitchlu et al., 2024); together these data mark it as a direct catalyst for severe post-chemotherapy adverse events and unplanned treatment cessation. Accordingly, the XAI system must not blindly accept electronic health record (EHR) baseline GFR values derived from the Cockcroft-Gault formula; instead, the algorithm should prioritise and assign the highest weight to the true GFR calculated via the CKD-EPI 2021 combined equation. Fusing this advanced biomarker with the underlying AI logic eliminates baseline biases attributable to sarcopenia and materially deepens the onconephrological rigour of the risk-stratification layer.

Beyond static baselines, a robust XAI system must incorporate a dynamic deconfounding architecture that establishes a real-time, bidirectional data channel with the EHR. When the algorithm detects the concurrent use of negative chronotropic agents (e.g., beta-blockers or ACE inhibitors), it must automatically recalibrate its warning thresholds and down-weight heart rate variability (HRV) parameters, whose informativeness is pharmacologically blunted under such therapy. The system must further embed basic electrocardiographic (ECG) filtering: upon detecting atrial fibrillation, frequent premature ventricular contractions (PVCs), or paced rhythms, it should suppress the now-invalid HRV module and pivot to other high-confidence metrics: body temperature, respiratory-rate micro-fluctuations, and the Cystatin C–anchored renal-function trajectory described above. This adaptive suppression-and-substitution logic ensures that the proposed digital safety net remains resilient to the pharmacological and arrhythmic complexities of real-world oncology practice, rather than degrading into a source of confounded, low-specificity alerts.

For example, when the system pushes a critical alert to the physician’s terminal, the XAI does not simply output “High Risk of Sepsis”. Instead, it gives a clear clinical explanation: “This 68-year-old male patient is on day 10 after chemotherapy, entering the neutrophil nadir indicated by the Weibull distribution. Over the past 4 h, the system detected an anomalous HRV decline with low-grade tachycardia. Based on stable weight and daily fluid intake, the algorithm excluded severe gastrointestinal dehydration as the cause of the tachycardia. The integrated probability of an impending neutropenic sepsis event is 85%.”

This transparent reasoning, grounded in evidence-based medical logic, filters out confounding factors such as dehydration and markedly reduces alarm fatigue. It also builds physician trust in the AI, which lets clinical teams start pre-emptive interventions before irreversible tissue ischemia and shock (Yang et al., 2020).

## 4 Future perspectives: pre-emptive therapeutics and policy reshaping

As cVSM and artificial intelligence continue to advance, their clinical value extends well beyond single early-warning alerts. We argue that the ultimate role of cVSM is to reshape the management of oncological complications and to move the point of care forward. As a basis, this shift can drive changes in health policy, insurance payment systems, and clinical-trial design.

### 4.1 Technological integration: establishing seamless outpatient monitoring and POCT closed-loops

Future digital oncology infrastructure will reach further into the patient’s home. This will enable closed-loop integration between cVSM and home-based point-of-care testing (POCT). In pursuing this vision, however, we must keep a rigorous and objective view of the regulatory landscape and the clinical applicability of new medical devices.

At present, microfluidic and optical colorimetry technologies, such as the HemoCue WBC DIFF, can provide total leukocyte counts and differentials within 1 min. Yet these devices are mainly approved by the FDA for POCT in professional healthcare facilities. Their move toward at-home self-testing is still being explored through strict usability evaluations. Importantly, they require patients to perform finger pricks to collect capillary blood. In chemotherapy patients with severe thrombocytopenia, this minimally invasive step carries a real risk of bleeding, causes pain, and may reduce long-term adherence (Lohman et al., 2018).

Consequently, the evolutionary focus of cutting-edge technology is rapidly shifting toward completely non-invasive monitoring dimensions. For instance, PointCheck (developed by Leuko Labs), a non-invasive optical white-blood-cell monitoring system originating from the Massachusetts Institute of Technology (MIT), quantifies circulating leukocytes at the nailfold capillary bed. This is achieved by non-invasively imaging the patient’s fingernail, utilizing specific wavelengths of light to penetrate the microcapillaries of the nail bed, and integrating deep learning with computer vision algorithms (Bourquard et al., 2018). This design completely eliminates the pain of blood drawing and the associated risk of opportunistic infections. Such devices are currently undergoing large-scale clinical registration trials under the strict regulatory framework of the FDA. Critically, feasibility is no longer solely an optical question but also one of human–computer interaction: a multicenter mixed-methods usability study of PointCheck in 154 untrained, first-time users operating the device in a simulated home environment reported a mean System Usability Scale (SUS) score of 86.1 (placing it within the top 10th percentile of systems evaluated by the SUS), with 70.1% of participants exceeding the 80.8 “excellent” usability threshold and 93.4% reporting high confidence in use, thereby supporting the feasibility of zero-barrier, high-frequency home-based immune monitoring, including in older patients (Lamaj et al., 2022). These successive advances in the underlying hardware and human-factors evidence base are paving the way for painless, high-frequency, home-based immune monitoring.

Under this model, when XAI-powered cVSM wearables detect subtle early hemodynamic changes or sustained low-grade fever, the system does more than alert the medical team. It also pushes instructions to the patient’s smart terminal, prompting an immediate neutrophil test with the next-generation POCT devices described above. The results are then transmitted in real time to the hospital cloud through the Internet of Things (IoT).

This dual closed-loop system, employing continuous vital sign monitoring for early detection and immediate biochemical verification as a diagnostic basis, overcomes the spatiotemporal constraints inherent in traditional episodic healthcare delivery. This synergy between cVSM early warning and biomarker-based confirmation will soon become a clinical reality (Verma et al., 2021). Medical teams will be empowered to acquire real-time, precise data regarding the patient’s ANC and systemic physiological status while the patient remains comfortably at home. Ultimately, this facilitates the genuine remote advancement and decentralization of high-tier medical resources.

### 4.2 Clinical intervention: the precise implementation of the “pre-emptive therapeutics” paradigm

Building on this integrated digital closed-loop, the management of infectious complications in NSCLC will undergo a qualitative shift: from reactive rescue to pre-emptive therapeutics. Pre-emptive therapeutics means interrupting the pathophysiological cascade before overt septic shock or organ failure. It relies on the microscopic deterioration trajectories captured by high-dimensional data together with immediate blood indices (Banerjee et al., 2021).

Once the multimodal monitoring system confirms an impending critical risk, the clinical team can rapidly issue online intervention directives, potentially dispatching emergency medications directly to the patient’s home or a nearby community clinic via telemedicine channels. For individuals whom the algorithm predicts to be at an exceptionally high risk of myelosuppression, but who have not yet developed a severe systemic infection, clinicians can precisely guide the prophylactic or intensified administration of granulocyte colony-stimulating factor (G-CSF) to reduce the risk of febrile neutropenia and accelerate hematopoietic recovery (Gyawali et al., 2026).

Conversely, for patients whose XAI early warning indicates that the sepsis cascade has already been initiated at the molecular and endothelial levels (e.g., evidenced by a sudden drop in HRV accompanied by low-grade fever), clinical teams can bypass the protracted wait times of the emergency department. Individualized, pre-emptive oral broad-spectrum antibiotic therapy or very early intravenous hydration interventions can be initiated immediately.

This time-critical intervention moves the traditional “door-to-needle time”, usually measured from emergency-room arrival, to hours before overt clinical symptoms. Such a shift could sever the path from sepsis to irreversible multiple organ dysfunction syndrome (MODS) and reduce sepsis-related mortality to a minimum.

### 4.3 Policy reshaping and the evolution of reimbursement pathways

Any disruptive medical paradigm needs strong guidance from health policy and structural support from insurance payment systems. Global healthcare systems now face rising cost-containment pressure. Unplanned emergency department (ED) visits, prolonged intensive care unit (ICU) stays, and extended hospitalizations from chemotherapy-induced neutropenic sepsis consume large direct and indirect resources (Gudiol et al., 2021). The Enhancing Oncology Model (EOM) from the Centers for Medicare and Medicaid Services (CMS) names better outpatient management and fewer preventable emergency admissions as core goals for future oncology payment reform.

Global healthcare payers should therefore recognize and quantify the health-economic value of digital interventions in reducing catastrophic critical-care costs. Building on existing Remote Physiological Monitoring (RPM) and Remote Therapeutic Monitoring (RTM) billing frameworks, such as US CPT codes 99,453, 99,454, and 99,457, policymakers should add specific reimbursement provisions for oncology patients on high-emetogenic and high-myelosuppressive regimens.

Specifically, we recommend that insurance cover, independently and in full, the hardware and software leasing costs for cVSM systems, POCT consumables, patient digital-literacy training, and the clinical time for data interpretation and intervention during the absolute high-risk window (the 30 days after chemotherapy). Such value-based compensation is essential to encourage oncologists to adopt these life-saving digital technologies.

### 4.4 Calling for clinical trials centered on validating digital pathways

To speed the entry of this digital early-warning and intervention paradigm into mainstream guidelines, the clinical community should launch well-designed, large-scale, multicenter RCTs. Unlike traditional oncology drug-registration trials, these studies should adopt a Decentralized Clinical Trial (DCT) design. This approach uses remote electronic patient-reported outcomes (ePROs), wearable data acquisition, and mobile community phlebotomy services (Adesoy et al., 2023).

For trial endpoints, beyond traditional measures of chemotherapy tolerability, primary efficacy endpoints should include the unplanned ICU admission rate, the time-to-antibiotic intervention (shifting from door-to-needle time to alert-to-needle time), and the early mortality rate from severe adverse events.

As robustly demonstrated in the trials conducted by Basch et al., systematic remote symptom monitoring improves health-related quality of life and reduces emergency department utilization (Basch et al., 2022), and an earlier single-institution randomized trial reported a significant prolongation in overall survival (OS) with proactive electronic symptom self-reporting (Basch et al., 2017). Consequently, it is highly plausible that digital trials centered on cVSM and XAI frameworks will similarly yield definitive, large-scale prospective evidence demonstrating extended OS among patients with NSCLC.

## 5 Conclusion

Through in-depth mining and modeling of a large, multinational real-world cohort, this study delineates the toxicological complexity and mortality risk of cisplatin-based therapy for NSCLC. In particular, the very high reporting odds ratio (ROR 9.23) and the newly validated septic-shock signals in FAERS and JADER, together with the “early failure” pattern (β = 0.81) from the Weibull model, where 56.6% of adverse events fall within the first 30 days, provide strong empirical evidence of these threats. The course of neutropenic sepsis is typically silent, rapid, and lethal. Reliance on traditional “reactive” paradigms, driven by routine outpatient follow-up and emergency admissions, is inadequate for modern precision medicine and digital health.

cVSM, augmented by XAI-driven risk-stratification algorithms and integrated into a closed loop with non-invasive home POCT, offers a way to close outpatient monitoring blind spots and achieve pre-emptive therapeutics. We therefore urge influential policymaking bodies, such as the American Society of Clinical Oncology (ASCO) and the European Society for Medical Oncology (ESMO), to take a proactive, forward-looking stance. These digital continuous-monitoring technologies should be incorporated into future revisions of oncological care standards and clinical guidelines for febrile neutropenia. Only by integrating foundational digital technologies with top-tier guidelines can we build a responsive digital safety net for every severely immunosuppressed patient with NSCLC.

## Data Availability

The original contributions presented in the study are included in the article/Supplementary Material, further inquiries can be directed to the corresponding author.
