# Supplementary material for: Continuous multidimensional physiological monitoring redefines early warning and preemptive treatment for neutropenic sepsis following cisplatin chemotherapy in non-small cell lung cancer
Source: Front Pharmacol. 2026 Aug 21;17:1861637. doi: 10.3389/fphar.2026.1861637 (PMC13541713; doi:10.3389/fphar.2026.1861637)
Supplement: Supplementary file 1 [file Table1.docx]

Supplementary Table 1:

Two-by-two contingency table for disproportionality analyses.

|  | Target AEs | Other AEs | Total |
| --- | --- | --- | --- |
| Cisplatin | a | b | a+b |
| Other drugs | c | d | c+d |
| Total | a+c | b+d | a+b+c+d |

Abbreviation: AEs, adverse events; a, number of reports containing both the target drug and target adverse drug reaction; b, number of reports containing other adverse drug reaction of the target drug; c, number of reports containing the target adverse drug reaction of other drugs; d, number of reports containing other drugs and other adverse drug reactions.
