# Supplementary material for: Continuous multidimensional physiological monitoring redefines early warning and preemptive treatment for neutropenic sepsis following cisplatin chemotherapy in non-small cell lung cancer
Source: Front Pharmacol. 2026 Aug 21;17:1861637. doi: 10.3389/fphar.2026.1861637 (PMC13541713; doi:10.3389/fphar.2026.1861637)
Supplement: Supplementary file 3 [file Table3.docx]

Supplementary Table 3:

Signal strength of Cisplatin AEs across System Organ Classes (SOC) in the FAERS database.

| System Organ Class (SOC) | Case numbers | ROR(95%Cl) | PRR(χ2) | EBGM(EBGM05) | IC(IC025) |
| --- | --- | --- | --- | --- | --- |
| Blood and lymphatic system disorders* | 321 | 2.51 ( 2.23 - 2.82 ) | 2.33 ( 251.66 ) | 2.3 ( 2.09 ) | 1.2 ( 1.03 ) |
| Vascular disorders* | 129 | 2.16 ( 1.8 - 2.58 ) | 2.1 ( 74.53 ) | 2.08 ( 1.79 ) | 1.05 ( 0.79 ) |
| Gastrointestinal disorders* | 420 | 1.46 ( 1.32 - 1.62 ) | 1.39 ( 51.02 ) | 1.38 ( 1.27 ) | 0.47 ( 0.32 ) |
| General disorders and administration site conditions | 344 | 0.86 ( 0.77 - 0.96 ) | 0.87 ( 7.1 ) | 0.88 ( 0.8 ) | -0.19 ( -0.36 ) |
| Skin and subcutaneous tissue disorders | 48 | 0.3 ( 0.23 - 0.4 ) | 0.32 ( 75.18 ) | 0.32 ( 0.25 ) | -1.65 ( -2.07 ) |
| Injury, poisoning and procedural complications | 88 | 0.83 ( 0.67 - 1.02 ) | 0.83 ( 3.1 ) | 0.83 ( 0.7 ) | -0.26 ( -0.58 ) |
| Neoplasms benign, malignant and unspecified (incl cysts and polyps) | 137 | 0.6 ( 0.51 - 0.71 ) | 0.62 ( 34.16 ) | 0.62 ( 0.54 ) | -0.68 ( -0.93 ) |
| Nervous system disorders | 159 | 1.09 ( 0.92 - 1.28 ) | 1.08 ( 1 ) | 1.08 ( 0.94 ) | 0.11 ( -0.13 ) |
| Musculoskeletal and connective tissue disorders | 32 | 0.41 ( 0.29 - 0.58 ) | 0.41 ( 27.31 ) | 0.42 ( 0.31 ) | -1.27 ( -1.77 ) |
| Respiratory, thoracic and mediastinal disorders | 218 | 0.75 ( 0.65 - 0.86 ) | 0.77 ( 17.27 ) | 0.77 ( 0.68 ) | -0.38 ( -0.58 ) |
| Cardiac disorders* | 111 | 1.27 ( 1.05 - 1.54 ) | 1.26 ( 5.98 ) | 1.25 ( 1.07 ) | 0.33 ( 0.05 ) |
| Renal and urinary disorders* | 93 | 1.48 ( 1.2 - 1.83 ) | 1.47 ( 13.91 ) | 1.46 ( 1.23 ) | 0.55 ( 0.24 ) |
| Metabolism and nutrition disorders* | 140 | 1.3 ( 1.09 - 1.54 ) | 1.28 ( 8.9 ) | 1.28 ( 1.11 ) | 0.35 ( 0.1 ) |
| Infections and infestations* | 253 | 1.47 ( 1.29 - 1.67 ) | 1.43 ( 33.83 ) | 1.42 ( 1.27 ) | 0.5 ( 0.31 ) |
| Eye disorders | 9 | 0.27 ( 0.14 - 0.53 ) | 0.28 ( 17.1 ) | 0.28 ( 0.16 ) | -1.84 ( -2.75 ) |
| Ear and labyrinth disorders* | 17 | 2.38 ( 1.47 - 3.85 ) | 2.37 ( 13.13 ) | 2.33 ( 1.56 ) | 1.22 ( 0.53 ) |
| Hepatobiliary disorders | 21 | 0.32 ( 0.21 - 0.5 ) | 0.33 ( 29.35 ) | 0.33 ( 0.23 ) | -1.59 ( -2.21 ) |
| Investigations | 155 | 0.7 ( 0.59 - 0.82 ) | 0.71 ( 19.32 ) | 0.71 ( 0.62 ) | -0.48 ( -0.72 ) |
| Product issues | 1 | 0.77 ( 0.11 - 5.5 ) | 0.77 ( 0.07 ) | 0.77 ( 0.15 ) | -0.38 ( -2.43 ) |
| Psychiatric disorders | 38 | 0.94 ( 0.68 - 1.3 ) | 0.94 ( 0.12 ) | 0.95 ( 0.72 ) | -0.08 ( -0.55 ) |
| Immune system disorders | 13 | 0.74 ( 0.43 - 1.28 ) | 0.74 ( 1.14 ) | 0.75 ( 0.47 ) | -0.42 ( -1.2 ) |
| Congenital, familial and genetic disorders | 3 | 0.46 ( 0.15 - 1.42 ) | 0.46 ( 1.94 ) | 0.46 ( 0.18 ) | -1.12 ( -2.57 ) |
| Surgical and medical procedures | 4 | 0.43 ( 0.16 - 1.14 ) | 0.43 ( 3.08 ) | 0.43 ( 0.19 ) | -1.22 ( -2.52 ) |
| Endocrine disorders | 3 | 0.09 ( 0.03 - 0.27 ) | 0.09 ( 29.32 ) | 0.09 ( 0.03 ) | -3.52 ( -4.96 ) |
| Pregnancy, puerperium and perinatal conditions | 1 | 3.55 ( 0.48 - 26.15 ) | 3.55 ( 1.76 ) | 3.45 ( 0.65 ) | 1.79 ( -0.33 ) |

Abbreviation: Asterisks (*) indicate statistically significant signals in algorithm; ROR, reporting odds ratio; PRR, proportional reporting ratio; EBGM, empirical Bayesian geometric mean; EBGM05, the lower limit of the 95% CI of EBGM; IC, information component; IC025, the lower limit of the 95% CI of the IC; CI, confidence interval; AEs, adverse events.
