# Supplementary material for: Continuous multidimensional physiological monitoring redefines early warning and preemptive treatment for neutropenic sepsis following cisplatin chemotherapy in non-small cell lung cancer
Source: Front Pharmacol. 2026 Aug 21;17:1861637. doi: 10.3389/fphar.2026.1861637 (PMC13541713; doi:10.3389/fphar.2026.1861637)
Supplement: Supplementary file 4 [file Table4.docx]

Supplementary Table 4:

Signal strength of Cisplatin AEs across System Organ Classes (SOC) in the JADER database.

| System Organ Class (SOC) | Case numbers | ROR(95%Cl) | PRR(χ2) | EBGM(EBGM05) | IC(IC025) |
| --- | --- | --- | --- | --- | --- |
| Gastrointestinal disorders* | 142 | 1.36 ( 1.14 - 1.63 ) | 1.32 ( 11.67 ) | 1.31 ( 1.1 ) | 0.39 ( -1.28 ) |
| Metabolism and nutrition disorders* | 106 | 1.98 ( 1.62 - 2.43 ) | 1.9 ( 44.99 ) | 1.86 ( 1.51 ) | 0.89 ( -0.78 ) |
| Renal and urinary disorders* | 55 | 1.34 ( 1.02 - 1.76 ) | 1.33 ( 4.39 ) | 1.31 ( 1 ) | 0.39 ( -1.28 ) |
| Blood and lymphatic system disorders* | 149 | 2.54 ( 2.13 - 3.03 ) | 2.36 ( 115.64 ) | 2.28 ( 1.91 ) | 1.19 ( -0.48 ) |
| Cardiac disorders | 31 | 0.72 ( 0.5 - 1.03 ) | 0.73 ( 3.27 ) | 0.73 ( 0.51 ) | -0.45 ( -2.12 ) |
| Respiratory, thoracic and mediastinal disorders | 136 | 0.39 ( 0.33 - 0.47 ) | 0.45 ( 114.69 ) | 0.46 ( 0.38 ) | -1.12 ( -2.79 ) |
| Investigations* | 243 | 2.51 ( 2.17 - 2.9 ) | 2.23 ( 169.16 ) | 2.15 ( 1.87 ) | 1.11 ( -0.56 ) |
| Infections and infestations | 87 | 1.1 ( 0.88 - 1.37 ) | 1.09 ( 0.72 ) | 1.09 ( 0.87 ) | 0.13 ( -1.54 ) |
| General disorders and administration site conditions | 49 | 0.81 ( 0.6 - 1.08 ) | 0.81 ( 2.13 ) | 0.82 ( 0.61 ) | -0.29 ( -1.96 ) |
| Vascular disorders* | 73 | 3.78 ( 2.95 - 4.85 ) | 3.63 ( 127.99 ) | 3.38 ( 2.64 ) | 1.76 ( 0.09 ) |
| Neoplasms benign, malignant and unspecified (incl cysts and polyps) | 22 | 0.34 ( 0.22 - 0.52 ) | 0.35 ( 27.43 ) | 0.36 ( 0.23 ) | -1.48 ( -3.15 ) |
| Nervous system disorders | 33 | 0.69 ( 0.49 - 0.98 ) | 0.7 ( 4.33 ) | 0.71 ( 0.5 ) | -0.5 ( -2.17 ) |
| Endocrine disorders | 56 | 0.59 ( 0.45 - 0.77 ) | 0.61 ( 14.96 ) | 0.61 ( 0.47 ) | -0.7 ( -2.37 ) |
| Hepatobiliary disorders | 25 | 0.38 ( 0.25 - 0.56 ) | 0.39 ( 25.2 ) | 0.39 ( 0.26 ) | -1.34 ( -3.01 ) |
| Psychiatric disorders | 5 | 0.8 ( 0.33 - 1.95 ) | 0.8 ( 0.24 ) | 0.81 ( 0.33 ) | -0.31 ( -1.99 ) |
| Skin and subcutaneous tissue disorders | 52 | 0.74 ( 0.56 - 0.98 ) | 0.75 ( 4.46 ) | 0.76 ( 0.57 ) | -0.4 ( -2.07 ) |
| Injury, poisoning and procedural complications | 7 | 0.31 ( 0.15 - 0.66 ) | 0.32 ( 10.45 ) | 0.32 ( 0.15 ) | -1.63 ( -3.31 ) |
| Ear and labyrinth disorders | 2 | 1.8 ( 0.43 - 7.45 ) | 1.8 ( 0.67 ) | 1.76 ( 0.42 ) | 0.81 ( -0.91 ) |
| Musculoskeletal and connective tissue disorders | 8 | 0.34 ( 0.17 - 0.67 ) | 0.34 ( 10.36 ) | 0.35 ( 0.17 ) | -1.53 ( -3.2 ) |
| Immune system disorders | 10 | 0.57 ( 0.3 - 1.06 ) | 0.57 ( 3.26 ) | 0.58 ( 0.31 ) | -0.8 ( -2.47 ) |
| Eye disorders | 6 | 0.78 ( 0.35 - 1.76 ) | 0.78 ( 0.35 ) | 0.79 ( 0.35 ) | -0.34 ( -2.02 ) |

Abbreviation: Asterisks (*) indicate statistically significant signals in algorithm; ROR, reporting odds ratio; PRR, proportional reporting ratio; EBGM, empirical Bayesian geometric mean; EBGM05, the lower limit of the 95% CI of EBGM; IC, information component; IC025, the lower limit of the 95% CI of the IC; CI, confidence interval; AEs, adverse events.
